# Dwarf Kingfisher-Inspired Bionic Flapping Wing and Its Aerodynamic Performance at Lowest Flight Speed

**DOI:** 10.3390/biomimetics7030123

**Published:** 2022-08-29

**Authors:** Mohd Firdaus Bin Abas, Balbir Singh, Kamarul Arifin Ahmad, Eddie Yin Kwee Ng, Tabrej Khan, Tamer A. Sebaey

**Affiliations:** 1 Department of Aerospace Engineering, Faculty of Engineering, Universiti Putra Malaysia, Serdang 43400, Selangor Darul Ehsan, Malaysia; 2 Department of Aeronautical and Automobile Engineering, Manipal Institute of Technology, Manipal Academy of Higher Education, Manipal 576104, Karnataka, India; 3 Aerospace Malaysia Research Centre, Faculty of Engineering, Universiti Putra Malaysia, Serdang 43400, Selangor Darul Ehsan, Malaysia; 4 School of Mechanical and Aerospace Engineering, College of Engineering, Nanyang Technological University, 50, Nanyang Avenue, Singapore 639798, Singapore; 5 Department of Engineering Management, College of Engineering, Prince Sultan University, Riyadh 11586, Saudi Arabia; 6 Department of Mechanical Design and Production Engineering, Faculty of Engineering, Zagazig University, Zagazig 44519, Sharkia, Egypt

**Keywords:** Kingfisher-inspired wing, flapping wing, kinematics, wingbeat frequency, bionic

## Abstract

This paper aims to understand the aerodynamic performance of a bio-inspired flapping-wing model using the dwarf Kingfisher wing as the bionic reference. The paper demonstrates the numerical investigation of the Kingfisher-inspired flapping-wing followed by experimental validation to comprehend the results fully and examine the aerodynamic characteristics at a flight velocity of 4.4 m/s, with wingbeat frequencies of 11 Hz, 16 Hz, and 21 Hz, at various angles of rotation ranging from 0° to 20° for each stroke cycle. The motivation to study the performance at low speed is based on lift generation as a challenge at low speed as per quasi-steady theory. The temporal evolution of the mean force coefficients has been plotted for various angles of rotation. The results show amplification of the maximum value for the cycle average lift and drag coefficient as the rotation angle increases. The history of vertical force and the flow patterns around the wing is captured in a full cycle with asymmetric lift development in a single stroke cycle. It is observed from the results that the downstroke generates more lift force in magnitude compared to the upstroke. In addition to the rotation angle, lift asymmetry is also affected by wing–wake interaction. Experimental results reveal that there is a stable leading-edge vortex developed in the downstroke, which sheds during the upstroke. An optimum lift and thrust flapping flight can be achieved, with a lift coefficient of 3.45 at 12°. The experimental and parametric study results also reveal the importance of passive rotation in wings for aerodynamic performance and wing flexibility as an important factor for lift generation.

## 1. Introduction

The demand for micro-aerial vehicles (MAVs) has grown at a rapid pace. Building an MAV presents its own set of challenges since its small size usually requires a short wingspan, which means that for flapping-wing MAVs, a single flapping period can only produce small lift and thrust force values. As a result, to maintain an effective flapping flight at a low Reynolds number, such as between 𝒪(10^3^) and 𝒪(15 × 10^3^), a particular MAV will have to take advantage of complex airflows through wake capture, one of several techniques. For even an ultra-small insect-based flapping, a deep understanding of unsteady aerodynamics is necessary. For example, mosquitoes have slightly different aerodynamic characteristics, such as less dependence on usual leading-edge vortices, trailing edge vortices through wake capture, dynamic stall, and rotational drag. This is accompanied by very high flapping frequency and low stroke amplitude [1]. Power sourcing and autonomous control are the biggest challenges. The Reynolds number at which effective flight is allowed decreases as wing length decreases, increasing the complexity of the flapping mechanism of such species. One of the preliminary studies performed by Park et al. [2] shows how a biomimetic ornithopter can be designed efficiently to have a sustained and controlled flight. The majority of current research on bio-inspired robots looks at traits that are as similar to those of their natural counterparts. Researchers are also now looking into the impact of airframe reconfigurability on these flapping-wing MAVs, which have been tested successfully on traditional quad and hexacopters [3] and also two of the most important characteristics of a bird flapping wing: downstroke and spanwise twisting. The former is concerned with the efficiency of the flapping operation, while the latter is concerned with reducing drag and power consumption [4].

**Figure 1:**
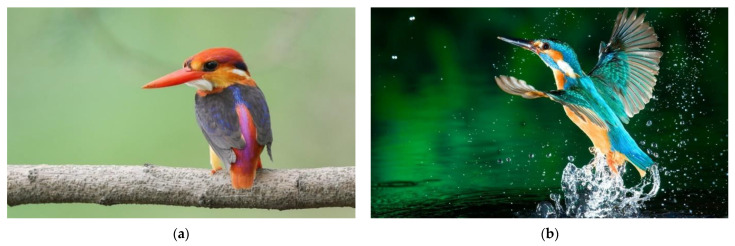
(**a**) Oriental Dwarf Kingfisher (*Ceyx erithacus*) [19]; (**b**) typical flying sequence of the kingfisher after hunting. Look at the downstroke with full wing spread (alula’s) to generate lift (Image free from www.wallpaperflare.com) (accessed on 14 April 2022).

There was a specific motivation behind choosing the dwarf kingfisher for this study. Most studies so far have focused either on large-sized ornithopter-type MAVs based on birds [5,6,7,8], unsteady and vibrational fluid-structure interaction on bats [9,10,11,12], or insect-type MAVs from dragonflies to bumblebees [13,14,15,16]. Only a few studies have focused on mid-range species between insects and birds, which have a variety of flight benefits as shown by Abas et al. [17,18]. These dwarf kingfishers have the ability to sustain hovering together with the regular head stability even if their body is oriented under the influence of the wind. This is because of their good aerodynamic ability and medium weight. This aids them in methodically capturing their prey. In comparison to other birds, they also have the least visual distortion. Additionally, these species make good use of their Alula when landing or grabbing prey at modest speeds. As a result, the focus of this research is based on the aerodynamics of a flapping wing model inspired by the Oriental Dwarf Kingfisher (*Ceyx erithacus*) as shown in Figure 1a, which is mostly found in Peninsular Malaysia. It has a smaller midrange wingspan of around 15 cm with shape like the one shown in Figure 1b and one of the few bird species that straddles the line between ornithopter and insect-like flight. Hummingbirds, for example, flap their wings in an insect-like manner (fast flapping), use their muscles properly and generate enough power to hover and fly.

This chosen species of Kingfisher has been observed to have a flight velocity range from 4.4 m/s to 8.8 m/s, limited by its small anatomy [20]. At a low flight velocity of 4.4 m/s, this dwarf Kingfisher can consistently fly above the surface of the water, awaiting opportunities to hunt its prey while conserving its energy for longer flight time. It can also boost its velocity up to 8.8 m/s to avoid threatening situations or escape from even larger predators. Although wing flexibility contributes to lift, it can reduce lift generation due to negative rotational lift and the adverse translation–rotation coupling effect after each stroke reversal [21]. The main contributions of this research are:

- To understand the aerodynamic mechanisms and their relationship to force production and aerodynamic efficiency on a nearly rigid flapping wing with the passive pitching kinematics [22] of a Kingfisher-inspired bionic wing.
- It is important to note that wing morphing (area change during the stroke), which is a crucial component of bird flight, is not taken into account here for simplicity, but flexibility (moderate stiffness) is used in the parametric study to see the effect on aerodynamic characteristics.
- The analysis is performed at a Reynolds number of 6024 at 4.4 m/s, which is the lowest flapping flight speed of the dwarf Kingfisher. The wing model’s flapping frequency is maintained at 11 Hz, the Kingfisher’s lowest flapping frequency.
- The study is also performed at 16 Hz and 21 Hz at the same flight speed all at different angles of rotation to observe the cycle average aerodynamic characteristics (mean lift and drag forces) of the wing model at higher wingbeat frequencies during flapping flight.
- The motivation for investigating low-speed performance is based on quasi-steady theory, which indicates that lift generation is a challenge at low flight speeds in hovering [23]. It is interesting how the dwarf kingfisher wing maintains lift at low speed.

The rest of this paper is organized as follows: wing morphology and design, kinematic modeling, and selection of an appropriate turbulence model for analysis, followed by numerical and experimental investigation to understand the aerodynamics behind dwarf Kingfisher flapping wing flight.

## 2. Numerical Methodology

For wing morphology and design, the numerical and experimental study were both executed using the bio-inspired wing as close as possible to the natural counterpart (Dwarf Kingfisher). The wing was designed using wing morphology i.e., real-time images and videos of flying Kingfishers were taken with a high-speed digital single-lens reflex (DSLR) camera equivalent to the Sony RX10 DSLR camera series to create the most realistic Kingfisher-inspired wing model possible.

**Figure 2:**
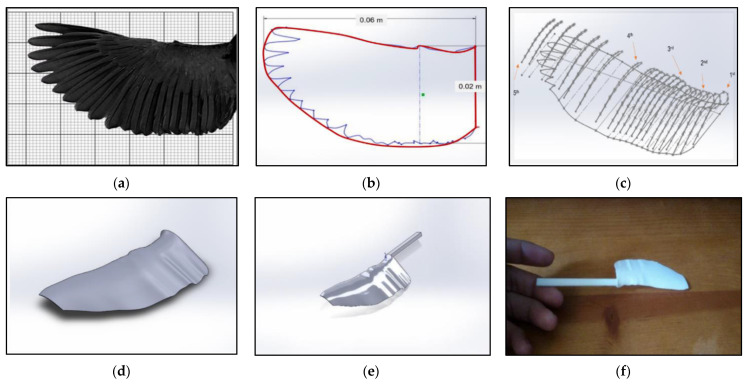
(**a**) Original wing ready for tracing process; (**b**) wing design after outline approximation; (**c**) framework of the profile-filling process; (**d**) and the finalized wing model; (**e**) complete corrugated (protrusion) CAD model; (**f**) 3D-printed wing model for experimental test rig.

To create a realistic three-dimensional Kingfisher-inspired wing model, the shape, angular dispersions, wing morphological characteristics like length, area, mean chord, etc. were analyzed using photo and video processing, including isometric imaging of photographs and stilled segments from videos of flying Kingfishers, a glimpse of which is shown in Figure 2a,b.

Several reference points were highlighted along the outline of the Kingfisher’s wing reference, and SolidWorks software was used to model the CAD geometry from those reference points. To offer the wing model a smooth finish, additional cross-sections (profiles) were created to fill the gaps between the five original cross-sections and relative to each original cross-section, as shown in Figure 2c,d. The complete corrugated (protrusion) CAD model and 3D-printed wing model for experimental test rig are shown in Figure 2e,f.

The process of tolerance and correction of the profile filling was made possible thanks to Oehme’s research paper on birds and generic wing design [24] (a wing design with commonly accepted cross-sections could be used as a guideline to design almost any kind of common bird’s wing). After all of the reference materials were processed, a three-dimensional smooth wing surface was created by combining all of the outlines and cross-sections. The Kingfisher’s wing was modeled as an isolated model to minimize design complexity and computational costs for both numerical and experimental validation. As mentioned earlier, the wing model was developed without considering the morphing characteristics, proper venation, and Alula. The wing model is thus obtained as a single three-dimensional smooth-surface object. The Kingfisher’s wing dimensions are mentioned in Table 1. The wing measurements were taken from a real Kingfisher and were corrected and improved with data from the identification guide created by Fry et al. [20] entitled Kingfishers, Bee-eaters, and Rollers.

### 2.1. Kinematic Modeling

**Figure 3:**
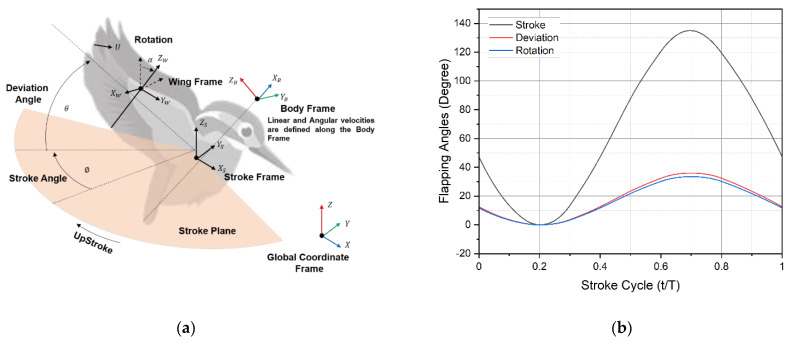
(**a**) The flying sequence of the kingfisher and detailed kinematic model; (**b**) standardized stroke cycle kinematics, flapping angles, rotation, stroke, and deviation versus the stroke cycle.

The morphological and kinematic models of the dwarf kingfisher are based on measurements of the Oriental Dwarf Kingfisher (*Ceyx erithacus*) wing described at the beginning of Section 2. Four coordinate systems are defined. The global fixed coordinates are X, Y, and Z, and the fixed local stroke plane coordinates are $X_{S}$, $Y_{S}$, and $Z_{S}$, with the origin at the base of the kingfisher wing. The $Y_{s}$ axis defines the spanwise variation and location. As shown in Figure 3a, the kingfisher’s attitude with respect to the stroke plane is defined by three Euler angles. These angles, which are determined using the axis local coordinate oriented with respect to the stroke plane, define motion attitudes, such as rotation, stroke, and deviation.

The wing motions are implemented using time histories of attitude angles that are rotated with respect to three different axes of the local coordinate system. As shown in Figure 3a, apart from global coordinate frame three more frames are defined; body ($X_{B}$, $Y_{B}$, and $Z_{B}$), stroke ($X_{S}$, $Y_{S}$, and $Z_{S}$) and wing ($X_{W}$, $Y_{W}$, and $Z_{W}$) coordinate frames. The wing stroke (Ø), deviation (θ), and rotation (α) angles are expressed in terms of the horizontal stroke plane frame. Their relationship with one full stroke cycle is shown in Figure 3b. Body linear and angular velocities are specified about the body frame [25]. The use of a predictive quasi-steady model approximation is one such best method to look into the unsteady aerodynamic forces and their influence, such as translation, rotation, translation–rotation coupling, and added-mass effect). We focused on translational and rotational forces in a most simple way.

### 2.2. Governing Equations and Numerical Flow Method

The governing equation of the flow is incompressible Navier-Stokes (N-S) Equations (1) and (2), in its non-dimensional format as

(1) $$St\frac{\partial u}{\partial \tau }+u\cdot \nabla u=-\nabla p+\frac{1}{Re}\nabla^{2}u$$

(2) ∇·u=0

where u and p are the non-dimensional flow velocity and pressure, respectively, τ is the non-dimensional time, and $St=\frac{c}{Ør_{2}}$ or $\frac{fA}{U_{ref}}\;$ and $Re=\frac{U_{ref}L_{ref}}{\nu }=\frac{4ØfR^{2}}{\nu \left(AR\right)}$ are the Strouhal number and Reynolds number, respectively. c is the mean chord length, ν is the kinematic viscosity of air, $r_{2}$ is the radius of gyration of the wing, A is peak-to-peak oscillation amplitude and stroke period T=1/f. Therefore, $U_{ref}=2fØr_{2}$. There are a few important factors to be taken into account while utilizing these equations in any kind of numeric analysis for example reference velocity, chord length, stroke frequency, amplitude, and position in a given time. The flapping motion source codes were created by using Equations (3)–(5) for three-dimensional angular flapping motions, as illustrated below. The relationship between all these flapping angles and their variation with the stroke cycle is shown in Figure 3b. Their parameterized form with third-order Fourier series are included in Equations (3)–(5).

Stroke angle or amplitude during the flapping motion:

(3) $$\phi \left(t\right)=\overset{n=3}{\underset{n=0}{\sum }}\left[\phi_{cn}\cos\left(2n\pi ft\right)+\phi_{sn}\cos\left(2n\pi ft\right)\right]$$

Wing deviation with respect to the stroke plane during the flapping motion:

(4) $$\theta \left(t\right)=\overset{n=3}{\underset{n=0}{\sum }}\left[\theta_{cn}\cos\left(2n\pi ft\right)+\theta_{sn}\cos\left(2n\pi ft\right)\right]$$

Wing rotation during the flapping motion:

(5) $$\alpha \left(t\right)=\overset{n=3}{\underset{n=0}{\sum }}\left[\alpha_{cn}\cos\left(2n\pi ft\right)+\alpha_{sn}\cos\left(2n\pi ft\right)\right]$$

where ϕ = positional angle, θ = elevation angle, α = angle of attack and n = integer and the factors $\phi_{cn},\;\phi_{sn}$ = Fourier coefficients of positional angle, $\theta_{cn},\theta_{sn}$ = Fourier coefficients of elevation angle, $\alpha_{cn},\alpha_{sn}$ = Fourier coefficients of the angle of attack.

### 2.3. Numerical Setup and Validation

The numerical analysis begins with the validation of the turbulence models, followed by mesh and time dependence tests. In both numerical and experimental studies, more emphasis is placed on the design and performance of the flapping wing model. The numerical performance is evaluated with an academic version of ANSYS-FLUENT using in-house UDFs written to accommodate the full kinematics of the wing flapping and aerodynamic models.

#### 2.3.1. Turbulence Model Validation

**Figure 4:**
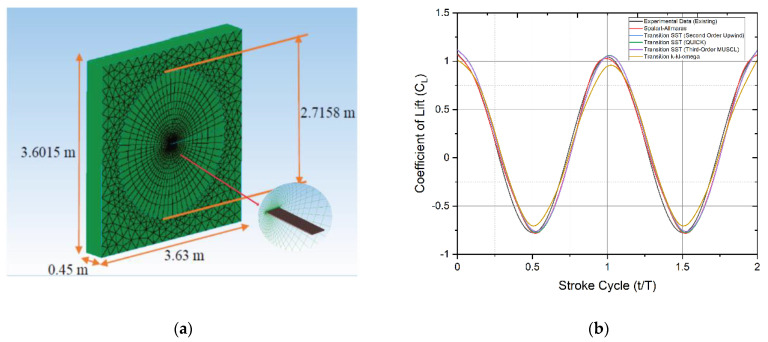
(**a**) Numerical setup for turbulence model validation; (**b**) lift distribution for a single stroke cycle for different turbulence models and their comparative study with available experimental data.

The minimum reference flight velocity of the dwarf Kingfisher is approximately 4.4 m/s, according to biological information on Kingfisher anatomy and flapping flight activity given by Fry et al. [20]. This reference flight velocity falls well within the transition fluid flow state for small birds. As a result of this transition fluid flow state, several transition turbulence models, primarily Transition SST and Transition k-kl-ω turbulence models, can be considered for the numerical approach. These turbulence models are varied along with various spatial discretization methods for accuracy refinement. They are also evaluated as part of basic numerical validities for this study in comparison with Lee et al. [26] experimental results based on the mean lift data, with an additional Spalart-Allmaras turbulence model for theoretical comparison and justification. Table 2 and Figure 4a display the wing model parameters and the modeled 3D numerical wing, respectively.

The mean lift distinction for a single stroke cycle between different turbulence models numerically evaluated and from Lee et al. [26] experimental results are shown in Figure 4b. When compared to the existing experimental results from the above study, the maximum mean lift value difference for Spalart-Allmaras, Transition SST (Second Order Upwind), Transition SST (QUICK), Transition SST (Third-Order MUSCL), and Transition k-kl-ω models is 3.858, 0.505, 0.654, 0.448, and 8.657 percent. When compared to the existing experimental results of the above study, the Spalart-Allmaras, Transition SST (Second Order Upwind), Transition SST (QUICK), Transition SST (Third-Order MUSCL), and Transition k-kl-ω models show 2.538, 0.421, 0.745, 1.197, and 5.280 percent difference in minimum lift value.

With less than a 3.0 percent difference between the mean lift values for one stroke cycle, the Transition SST (second order upwind) turbulence model shows the nearest and most consistent lift data when compared to the others, making it the most appropriate turbulence model for this numerical approach. This shear stress transport-based model can solve problems with both high and low turbulence and can explain the difference in these two turbulence magnitudes at the confluence between the wake behind an object and the free stream.

#### 2.3.2. Mesh and Time-Step Independence Tests

**Figure 5:**
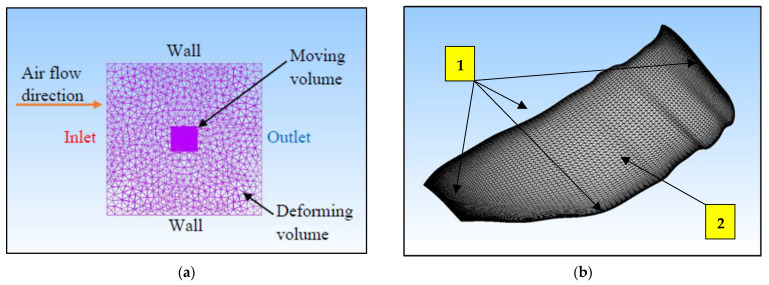
(**a**) Mesh modeling description of the wing for numerical analysis; (**b**) variation of lift coefficient with time step at various flapping frequencies.

**Figure 6:**
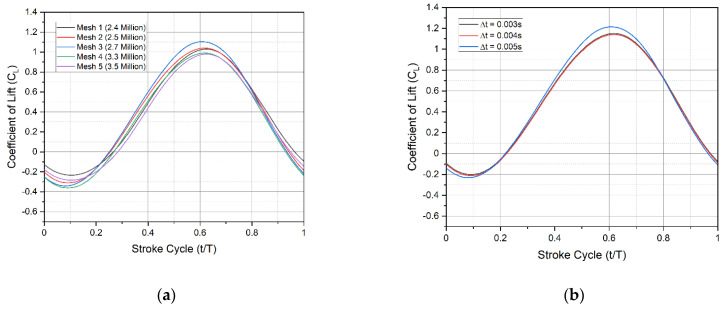
(**a**) Grid independence study and validation for a number of cells with normalized vertical force as a parameter of study for each stroke cycle; (**b**) time-step independence study and temporal evolution of mean lift as a parameter of study for different time-step in each stroke cycle.

The grid convergence study was carried out and repeated five times, yielding five different mesh densities until an appropriate mesh configuration was found. The cell counts of the five generated mesh configurations are approximately 2.4, 2.5, 2.7, 3.3, and 3.5 million, respectively. The mesh structure and overall mesh domain of the Kingfisher-inspired flapping-wing model used in this numerical study are shown in Figure 5a. High-resolution finer mesh is used near the edges (marked no. 1) of the wing model with overall structural mesh formation on the surface (marked no. 2) as shown in Figure 5b. To generate a high-accuracy and high-performance mesh configuration, each mesh configuration has been numerically investigated, and the respective force for each mesh configuration is evaluated for one full flapping cycle as presented in Figure 6a.

This convergence test was carried out at a speed of 4.4 m/s, a flapping frequency of 11 Hz, and at 4° angles of attack. The mesh configuration in the deforming volume region will deform in response to the flapping motion of the wing model boundary and the moving volume region. Mounting CFD codes onto the moving boundary and its adjacent volume allows the flapping motion to be executed through a sequence of these codes. The flapping motion source codes were created by using Equations (3)–(5) for angular flapping motions and simultaneously initiating all of the equations to complete the requisite 3D flapping wing pattern.

In comparison to mesh 5 with a 3.5 million-cell count, mesh 4 with a 3.3 million-cell count was chosen as the most appropriate mesh configuration to continue with the numerical investigations in this study because it consistently has a mean lift value difference of approximately 3.0 percent. As a result, the mesh with 3.3 million cells adheres to and achieves the best mesh density balance between simulation accuracy and computational cost. With a decrement of 0.001 s from 0.005 to 0.003 s, three different time-step sizes were introduced as shown in Figure 6b.

This time-step independence test was performed at 4.4 m/s flight velocity, 11 Hz flapping frequency, and 4° angles of attack. Figure 6b shows that the temporal evolution of mean lift versus the one full stroke cycle calculated with time step Δt = 0.003 s is almost equivalent to the results obtained with time steps Δt = 0.004 s and Δt = 0.005 s. As a result, the time step Δt = 0.003 s with moderate $C_{L}$ is used in all simulations.

## 3. Experimental Investigation and Setup

**Figure 7:**
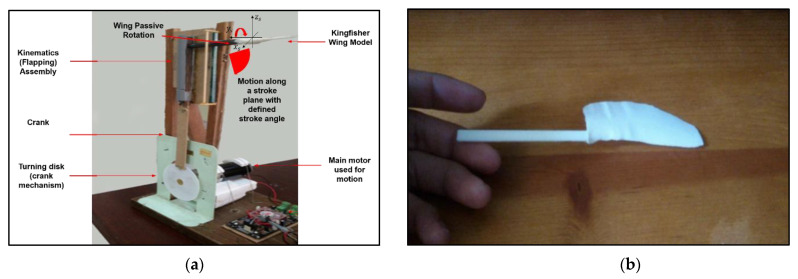

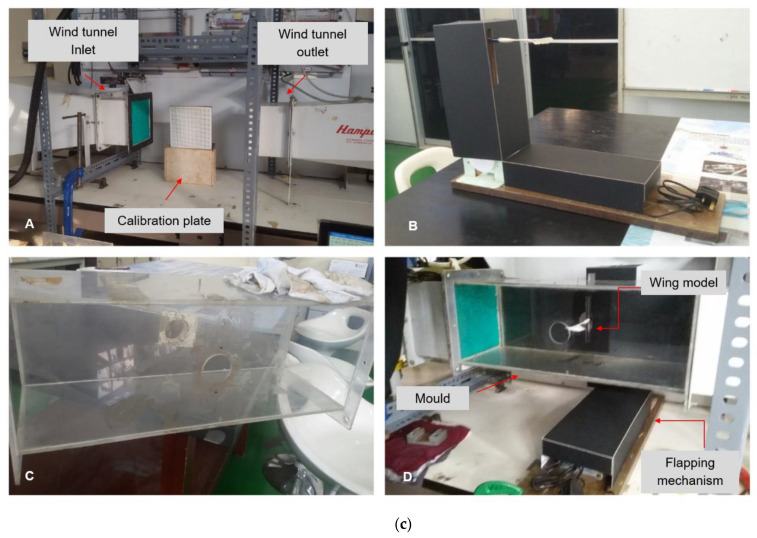
(**a**) Flapping Wing experimental test rig; (**b**) 3-D printed wing model product for experimental run; (**c**) details of the experimental test rig (flapping mechanism test setup); experimental setup consists of (**A**) PIV measurement area calibration; (**B**) completed flapping mechanism with mounted wing model, (**C**) test rig, and (**D**) completed PIV experiment assembly.

An experimental study was performed to ensure the validity of the numerical setup used here to investigate about aerodynamic characteristics of a dwarf Kingfisher-inspired flapping-wing model. The experiment aimed to provide appropriate evidence to support the numerical investigation used in this study. The flapping wing model was tested in a low-velocity wind tunnel equipped with a particle image velocimetry (PIV) system to analyze the velocity profile generated by the wing model’s vertical flapping motion. The numerical-experimental validation process needed data on velocity contours and vector plots. For simplicity, ease of handling, and cost efficiency, the validation test was carried out under the conditions described in Table 3. As shown in Figure 7a,b, the corrugated wing model made before for numerical calculations is 3D printed for experimental study. Figure 7c shows the flapping wing mechanism experimental test rig in detail. The test segment measures 0.3 m × 0.3 m × 1.0 m. Once the flow speed increased to 4.4 m/s, a smoke screen was injected into the test portion of the wind tunnel. The 2D PIV setup from Dantec Dynamics at the Aerodynamics Laboratory of Faculty of Engineering, Universiti Putra Malaysia is used for experimental test rig and analysis as shown in Figure 7c—Figure 7A–D.

The New Wave Gemini laser projector was strategically placed on top of the test section to create a laser sheet cross-section, allowing the cross-correlation Kodak ES 1.0 digital camera to capture the smoke-injected airflow particles moving past the flapping-wing model. The motor used was adjusted with constant motion (rotation) to maintain the flapping frequency of 11 Hz. To effectively capture every quarter cycle of a complete flapping-wing cycle and its respective airflow velocity profile over many flapping cycles until the end of the experimental run, the rate of laser projections and camera shots were set to 500 repetitions and 2 sets of 50-image bursts, respectively.

The experiment was carried out three times to ensure that the experimental setup could consistently produce the same velocity profile results. The PIV system’s computer, which was rigged with LaVision’s hardware and onboard PIV image analyzer software, processed and saved all of the velocity profile data.

## 4. Results and Discussion

In this research, a numerical investigation was performed on the Kingfisher-inspired flapping-wing model, with a Reynolds number of 6024 at 4.4 m/s, at 11 Hz, 16 Hz, and 21 Hz flapping frequency. It was carried out using a 32 GB Windows workstation platform (8 cores, 16 threads). The average runtime per parametric analysis was around 5 h, with an average runtime of nearly 1.5 h per complete cycle. The various angles of rotation (0° to 20° with 4° increments) were used to observe the aerodynamic characteristics (lift and drag forces) of the wing model during the flapping flight. This was followed by a thorough experimental study of the physical model.

### 4.1. Experimental Analysis and Study

**Figure 8:**
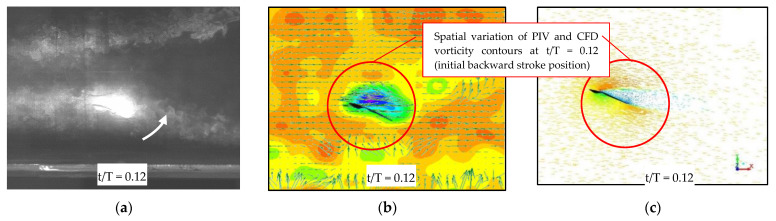
(**a**) Flapping of Kingfisher-inspired wing during experimentation (*z*-axis); (**b**) filtered velocity vector field with scalar map at the background, obtained from the 2D-PIV setup at t/T = 0.12; (**c**) CFD velocity vector field at t/T = 0.12.

**Figure 9:**
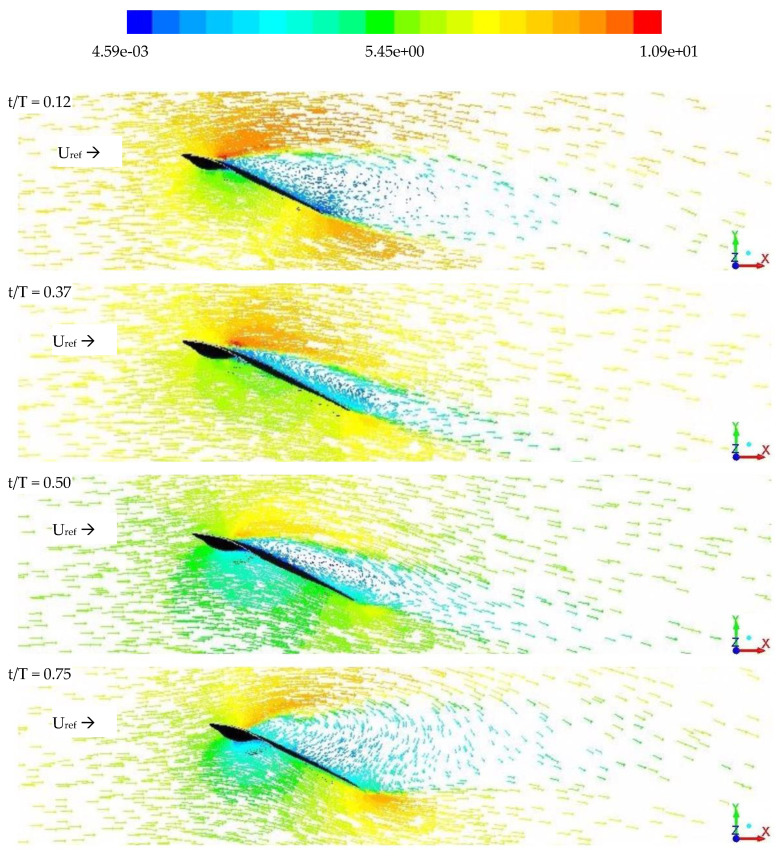
Vector flow field during the flapping motion (*z*-axis); left-to-right it shows the flow field vector plot during the moment of one full cycle (contours of motion during upstroke to downstroke at particularly chosen stroke cycle from t/T = 0.12, t/T = 0.37, t/T = 0.37, t/T = 0.75 at 20° AoA).

**Figure 10:**
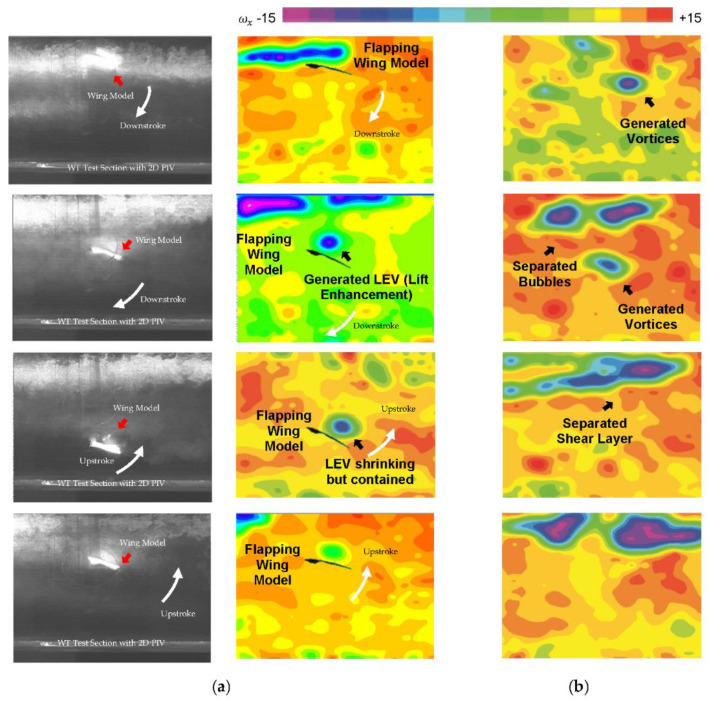
(**a**) One sample of the motion of the wing model along the stroke plane in the wind tunnel with PIV setup; (**b**) the formation of vortices particularly for lift enhancement, secondary separation bubbles, and separated shear layers shows an extremely unsteady environment although the wing can maintain the lift during flapping motion.

The aerodynamic force-time histories and flow fields obtained from experimental tests and CFD simulations are discussed in this section. The experimental force measurements and PIV flow field data are primarily used to study the kingfisher-inspired flapping wing’s predicted aerodynamic features. Figure 8a–c shows the comparison of velocity vector flow field obtained from PIV and CFD. Figure 9 measures and predicts the vector flow field from the numerical analysis for one full stroke cycle (a) t/T = 0.12, (b) 0.37, (c) 0.50, (d) 0.75) at a rotation angle of around 20° used for aerodynamic (lift and drag) force-time histories, clearly demonstrating flow separation at a later stage of flapping. Both the experiments and the numerical analysis show that there is a vortex generation that is akin to a combination of a leading-edge and bounded vortex, the energy of which is used to maintain lift during the downstroke. During the downstroke, the leading-edge vortices (LEV) near the wing root in Figure 10a,b is tightly formed and bonded to the wing. When compared to one other, both the CFD solution and the PIV were able to precisely estimate the LEV size and placement. The LEV has ruptured in Figure 10b, becoming more dispersed and separating from the wing surface. The production of vortices, secondary separation bubbles, and separated shear layers during flight indicates an exceedingly unsteady environment. The formation of strong stall related vortices, secondary separation bubbles, and separated shear layers shows an extremely unsteady environment, but the wing maintains the lift during the flight.

It is worth noting that the LEV in Figure 10b has shrunk in size and is no longer attached to the wing. A smaller secondary LEV has taken shape near the leading edge and a separated LEV, close to the wing surface. It is also observed that the increase in the angle α affects both the mean lift and drag values during the flapping flight. The 16° angle-of-attack shows the most variations in mean lift and drag values. This promotes a higher value of thrust at the expense of a reduced lift. Depending on the situation, a Kingfisher can freely maneuver its wing’s angle-of-attack to suit both a high lift and low drag leisure flapping flight or a high thrust flapping flight with significant lift, though the latter will not last this maneuver requires an enormous amount of energy and will leave the bird exhausted before long. Note that a 16° angle-of-attack flapping flight is the least effective flapping flight.

According to the experimental results shown in Figure 10a, the flow field around the wing generates a significant vortex upstream and a separation region far away from downstream on the wing. As the angle of attack increases, a strong vortex region near the trailing edge forms, the energy of which can be utilized further. The experimental analysis clearly shows the importance of making wing passive rotation necessary if bio-inspired imitation is to be successful in flight. In a stroke plane, the wing motion from upstroke to downstroke excites the layer of shear separation at the leading edge, which creates a form of Von Karman Vortex Street.

Therefore, aeroelastic customization is required to investigate further wing performance by controlling the flow using the passive rotation of the model wing. This is required for the fabrication of bio-inspired flying robotic models and compelled the authors to conduct additional parametric studies into the role of wing flexibility over rigidity.

### 4.2. Lift and Drag with Stroke Cycle in a Power Flight

**Figure 11:**
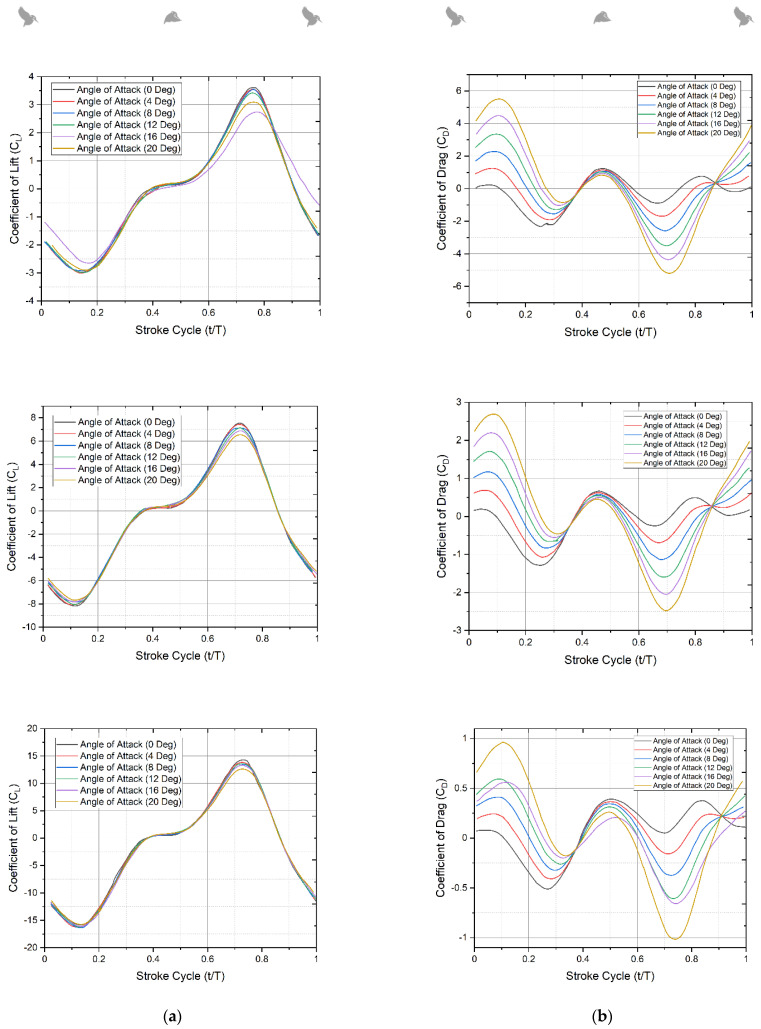
(**a**) Development of lift force during each stroke ($C_{L}$ v/s t/T) at 4.4 m/s at f = 11 Hz; Lift force during each stroke ($C_{L}$ v/s t/T) at 4.4 m/s at f = 16 Hz and lift force during each stroke ($C_{L}$ v/s t/T) at 4.4 m/s at f = 21 Hz; (**b**) drag force during each stroke ($C_{D}$ v/s t/T) at 4.4 m/s at f = 11 Hz; drag force during each stroke ($C_{D}$ v/s t/T) at 4.4 m/s at f = 16 Hz and drag force during each stroke ($C_{D}$ v/s t/T) at 4.4 m/s at f = 21 Hz. Forces are measured at different AoA ranging from 0° to 20°.

The simulation results from the CFD study are used in this part to evaluate the differences in aerodynamic force production and flow physics. The lift profile produced by all angles of attack studied demonstrates the same lift production cycle during power flight. Figure 11a,b depict the variance in instantaneous mean lift and drag force during one flap cycle for the three translational pitch scenarios at three different flap frequencies of 11 Hz, 16 Hz, and 21 Hz. Figure 11a,b show that the mean values of lift and drag rise as the flapping frequency increases, particularly during the forward stroke. The aerodynamic lift produced is positive for the majority of the stroke cycle in all scenarios with varying flapping frequency. Negative lift is created at the stroke reversal moments (t/T 0.0, 0.8, and 1.0) because of the delayed rotation of the specified pitch kinematics.

Figure 11b depicts the variation in drag for the three flapping frequencies and different angle instances across a stroke cycle. As anticipated, the peak amplitude of the drag force increases as the rotational angle increases. The peak in drag force occurs at the mid-forward and mid-backward strokes in all cases. It is to be noted that mean drag increases at t/T 0.1 and 0.45, and drag force is proportional to the square of rotation speed. The majority of the stroke cycle has a positive peak for mean lift; however, there is some negative lift at stroke reversal.

Because of the delayed rotation kinematics inherent in the passive pitching mechanism used, this behavior in the lift force trend is expected as per the existing literature [22]. The projected instantaneous force magnitudes are also described in the literature as being within the ranges of uncertainty for the experimental results. Figure 11b depicts the variance in drag across one full stroke cycle. The temporal plots of the mean drag value show that the drag force acted in the positive and negative *x*-directions, respectively. Drag worked against the flapping action of the wings throughout the flapping cycle. During this motion cycle, the measured and anticipated drag force values followed the same trend in the instance of the highest mean drag for each angle of rotation and flapping frequency. The maximum drag peak of the CFD drag force-time history occurs at the mid-forward and mid-backward strokes. The considerable divergence in the peak magnitude of drag force is due to the difficulty in distinguishing the large inertial loads in the stroke plane from the aerodynamic loads produced by the wing.

Therefore, based on the numerical results from the simulations, contour plots, and experimental results, it can be concluded that there is an asymmetry in the force between upstroke and downstroke, and the concept of the circulation is measured by the vorticity field is the primary source of lift force estimation. It is also worth noting that Kingfishers produce significantly more lift on the downstroke than on the upstroke, due to the development of edge vortices during the downstroke. The sudden decrease in the lift after t/T = 0.8 might be due to a very high flow separation with an increase in drag.

### 4.3. Parametric Study Related to Flexible and Rigid Wings

**Figure 12:**
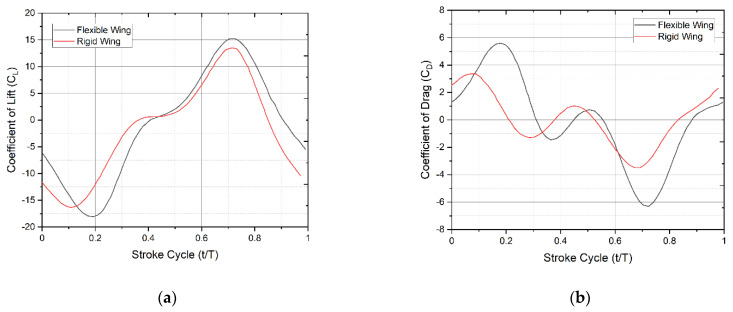
(**a**) Effect on lift production with respect to stroke cycle for corrugated rigid and flexible wing; (**b**) effect on drag with respect to stroke cycle for the corrugated rigid and flexible wing.

A parametric study was conducted to investigate the role of flexibility in lift augmentation. It was discovered that at a given stroke cycle, the rigid model’s lift is lower than that of the non-rigid model and that at certain times, the rigid model’s lift appears lower while the flexible model smoothly increases. Figure 12a,b show time histories of the mean lift and drag of deformable and rigid wings, respectively. One of the reasons for this might be the shedding of vortex being more in the rigid model than the flexible and the strong bond of the vortex generated, to the leading edge particularity at higher angles of attack.

Thus, wing flexibility increases lift and decreases drag by delaying the stall largely than the rigid model of the wing. The history of vertical force and the flow pattern around the wing is captured in a full stroke cycle. Results show asymmetric lift development with a single stroke cycle. It is observed from the results that the downstroke generates more lift force in magnitude compared to the upstroke. Analysis shows that, in addition to the rotation angle, lift asymmetry is also affected by wing–wake interaction. Experimental results reveal that there is a stable leading-edge vortex developed in the downstroke, which sheds during the upstroke.

**Figure 13:**
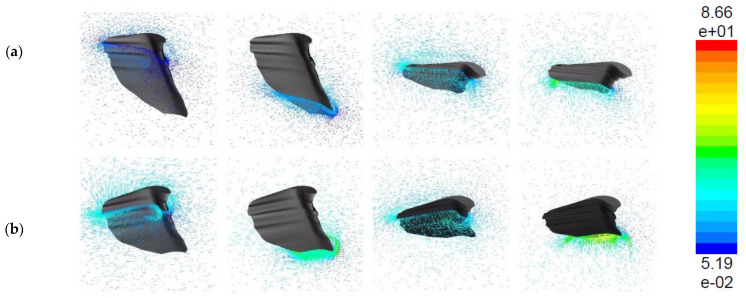
Vector plot of lift production with respect to stroke cycle for (**a**) corrugated rigid (**top**) and (**b**) flexible wing (**bottom**). The analysis is performed at 4.4 m/s, highest flapping frequency (21 Hz) at 12° angle of attack.

Until now, the analysis has been done under the assumption that the wing is rigid and no morphing is used. We conducted a brief study to see what happens when the wing is deforming; flexibility is introduced and compared to the rigid case. Wing deformation is maintained by spanwise modulation of the pitching angle, as detailed in [27]. To evaluate the effect of deformation, we replicate rigid wings by omitting the spanwise twist and comparing it to the deformable case. The rigid model’s pitching angle is kept the same as the wing’s base as described in [27]. The time histories of the mean lift and drag coefficients of both the deformable and rigid models are shown in Figure 12a,b. Although the rigid model’s mean lift is just 2% smaller than the deformable example, the time histories demonstrate distinct tendencies in the two situations. Figure 13a,b clearly shows the $C_{L}$ vector plot comparison between the rigid and the flexible wings respectively. The flexible wing generates more lift compared to rigid. This is due to the generation of leading vortices and their contribution as the t/T increases from left to right.

The mean lift of the rigid model is noticeably smaller than that of the deformable model throughout the cycle, whereas that of the flexible model increases continuously. Although we know from kinematics that the pitching angle nearly reaches neutral at each stroke reversal, increasing it at the start of the next stroke affects transient lift, implying that the flexibility of the wing structure and root connection can be detrimental to the lift generation of a flapping wing [27]. Although we have not computed the overall power consumption, it must be estimated to evaluate the energy recovery during flapping flight in the case of flexible wings.

## 5. Conclusions

There is no question that kingfisher is among the best flyers. The anatomy, kinematics, aerodynamics, and stability study of these creatures so far revealed the useful benefits of imitating such birds, creating drones, and using them for different applications. Here are the conclusions from this work:

1. Based on the findings, for this Kingfisher wing model, the mean lift and drag increase as the flapping frequency increases especially during the forward stroke. For all the cases with different flapping frequencies, the majority of the stroke cycle has a positive peak for mean lift; however, there is some negative lift at stroke reversal because of the delayed rotation kinematics.
2. Since this is a low-speed flight, both the experiments and the numerical analysis show that there is a vortex generation that is akin to a combination of the leading edge and bounded vortex, the energy of which is used to maintain lift during the downstroke. The LEV near the wing root is tightly formed and attached to the wing. The maximum amount of lift is generated during the downstroke-forward stroke.
3. The temporal evolution of the lift and drag coefficient has been plotted for 0, 4, 8, 12, 16, and 20-degree angles of attack. The results show amplification of the maximum value for the lift coefficient as the angle of attack is increased. However, the mean drag presents some variations in its temporal evolution. For that reason, a study of the time-averaged lift and drag coefficient using predictive quasi-stead or unsteady models depicting all the forces could be interesting in the future to see if the wing model produces thrust or not and how it evolves.
4. Passive rotation of wings is very important to implement in robotic models mimicking the bio-creature to enhance the wing aerodynamic performance. The lift generated by the rigid wing model was less than that of the flexible one. Therefore, flexibility is vital for the good aerodynamic performance of bionic wings and models.
5. It can be concluded that there is an asymmetry in the force between upstroke and downstroke, and the concept of the circulation is measured by the vorticity field is the primary source of lift force estimation. It is also worth noting that Kingfishers produce significantly more lift on the downstroke than on the upstroke, due to the development of edge vortices during the downstroke. The sudden decrease in the lift after t/T = 0.8 might be due to a very high flow separation with an increase in drag.
6. The flowfield was highly sensitive to variations in pitch angle, leading to commensurate changes in anticipated aerodynamic force trends. It is therefore good to look into the instantaneous aerodynamic power as an extension of this study in the future. Once the flow fields are obtained, it is also possible to report the instantaneous power, the aerodynamic and propulsive efficiency at each angle of incidence.

## Figures and Tables

**Table 1:** Kingfisher-inspired wing model specifications.

Specifications	Value
Span-wise length, R	0.06 m
$\mathrm{Mean}\;\mathrm{chord}\;\mathrm{length},\;c_{mean}=L_{ref}$	0.02 m
$\mathrm{Root}\;\mathrm{thickness},\;t_{root}$	0.0007 m
$\mathrm{Tip}\;\mathrm{thickness},\;t_{tip}$	0.0002 m
Aspect Ratio, AR	3.0 (General)

**Table 2:** Wing specifications for turbulence model validation reference to Lee et al. [26].

Specifications	Value
Span-wise length, R	0.18 m
$\mathrm{Chord-wise}\;\mathrm{length},\;c_{mean}=L_{ref}$	0.03 m
Thickness, t	0.0015 m
Aspect Ratio, AR	6.0
$\mathrm{Angle}\;\mathrm{of}\;\mathrm{Attack},\;\theta_{0}$	40°
Flapping amplitude, Ø	~120°
Flapping frequency, f	5 Hz
$\mathrm{Inlet}\;\mathrm{velocity},\;U_{\infty }$	6 m/s

**Table 3:** Experimental setup and specifications.

Specifications	Value
Angle of attack, α	20°
Flapping amplitude, Ø	~120°
Flapping frequency, f	11 Hz
$\mathrm{Airflow}\;\mathrm{velocity},\;U_{\infty }$	4.4 m/s
Laser emission	High
Number of laser projection	500 repetitions
Number of image burst	2
Number of images captured per burst	50

## Data Availability

Not applicable.
